# Supplementary figures and images for: Nutrition or nature: using elementary flux modes to disentangle the complex forces shaping prokaryote pan-genomes
Source: BMC Ecol Evol. 2022 Aug 16;22:101. doi: 10.1186/s12862-022-02052-3 (PMC9382767; doi:10.1186/s12862-022-02052-3)

A

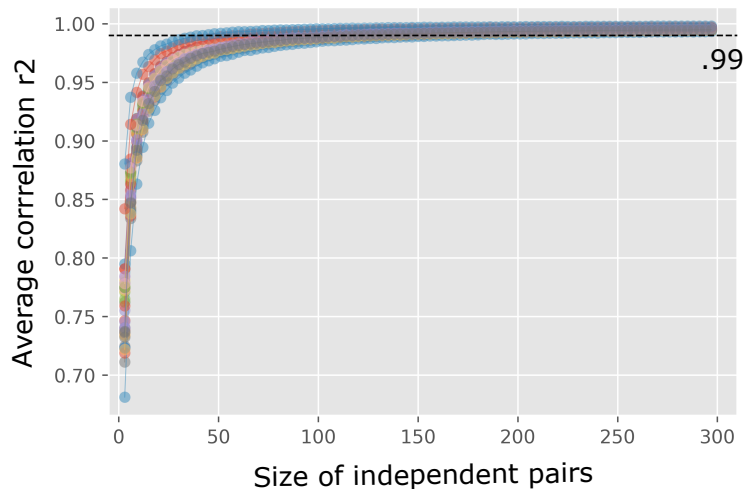

B

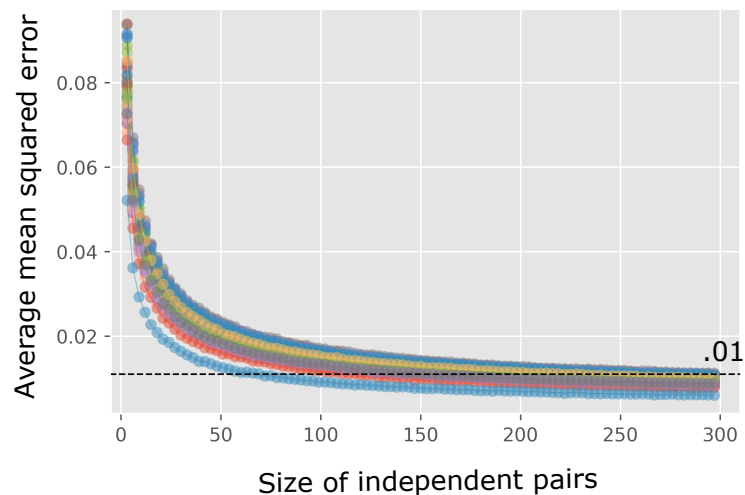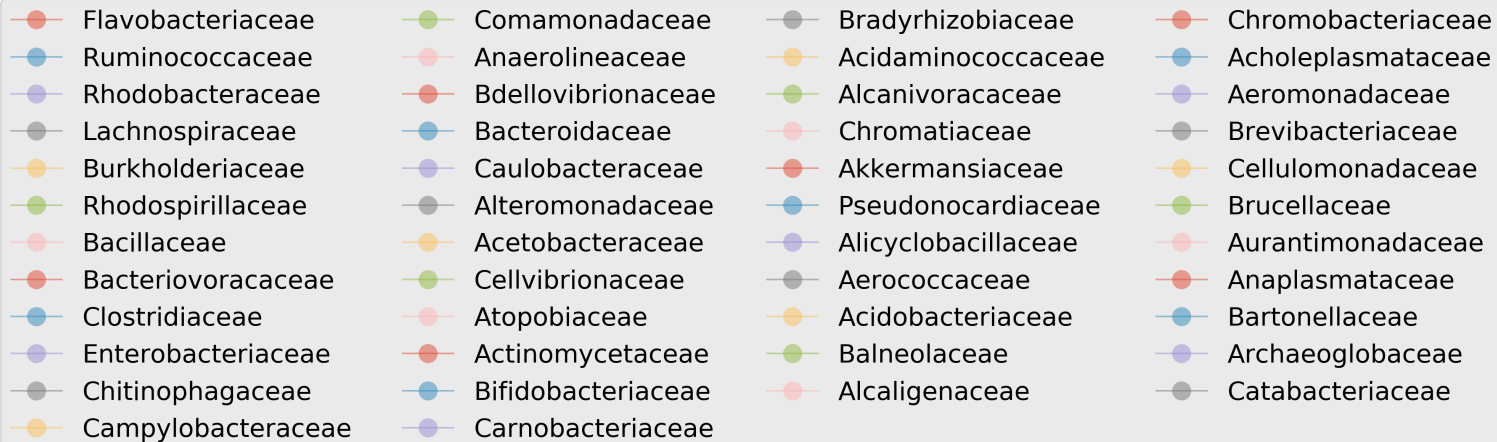

Supplement: Supplementary file 1 — Additional file 1: Figure S1. Convergence of the reaction frequencies of panEFMs sampled across 1000 virtual environments (Table S2) to an average. The frequency of reactions was obtained from two random-independent (non-overlapping) groups of panEFMs defined across the env [file 12862_2022_2052_MOESM1_ESM.pdf]

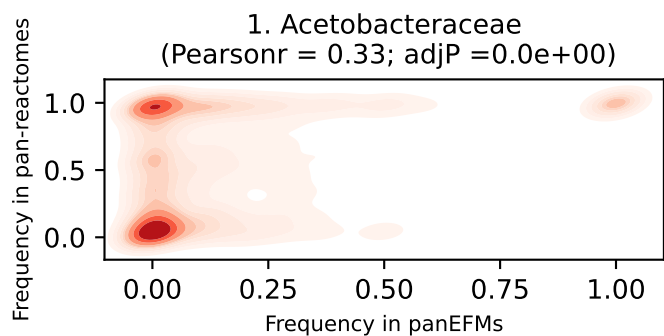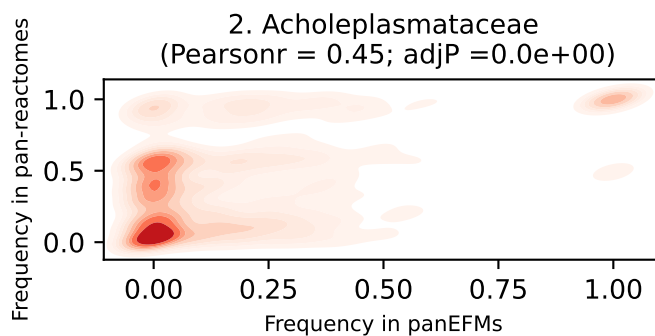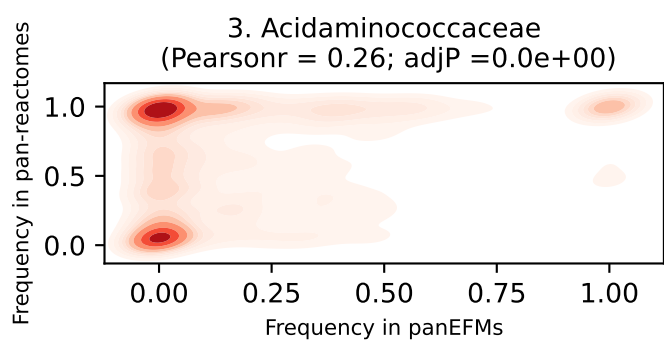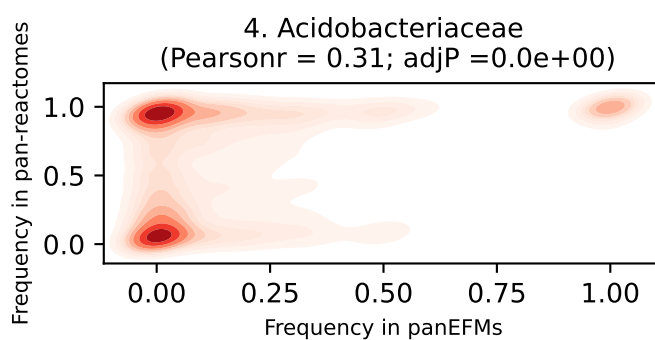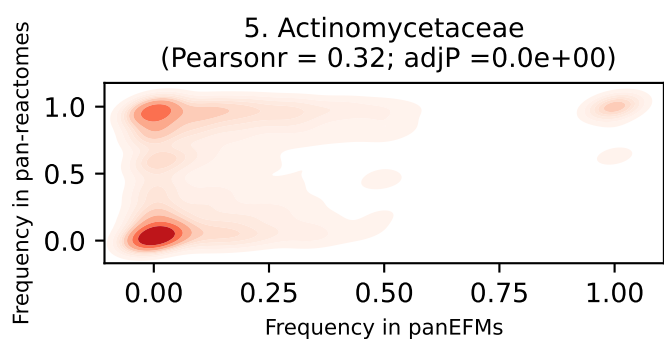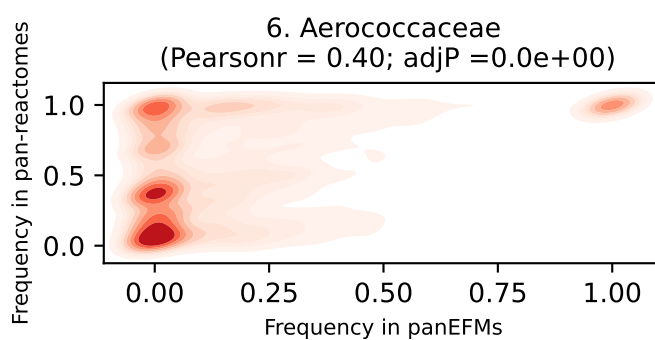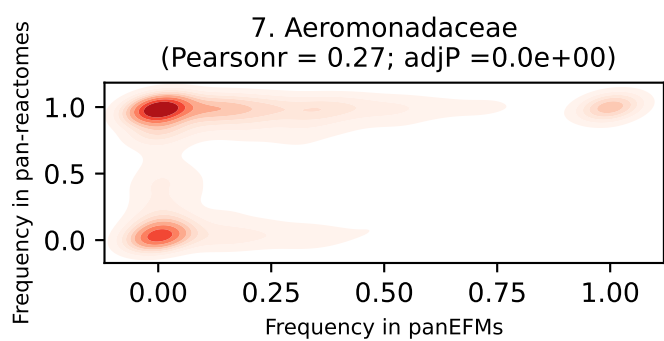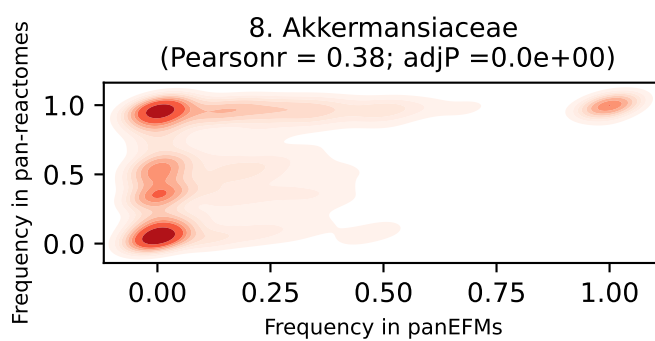

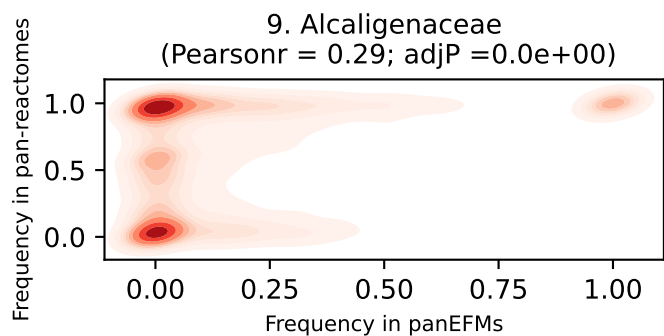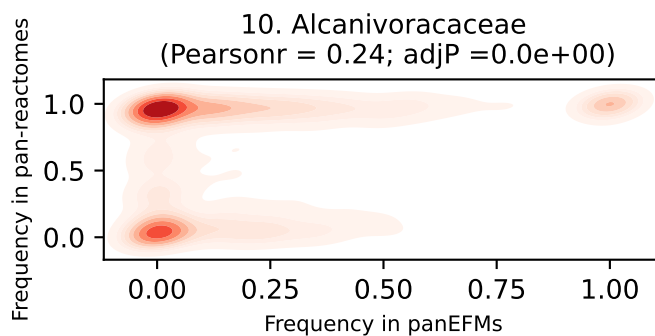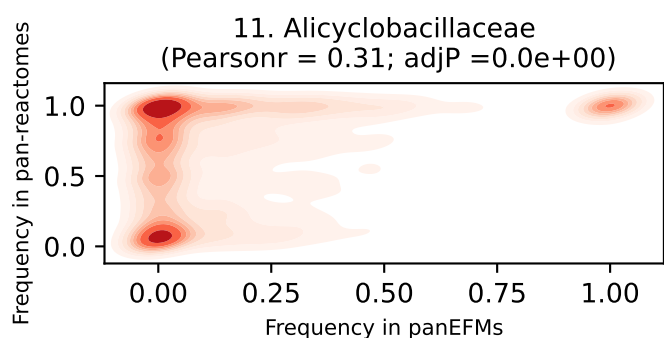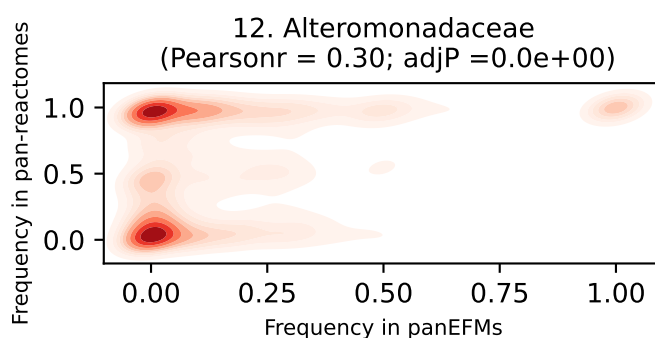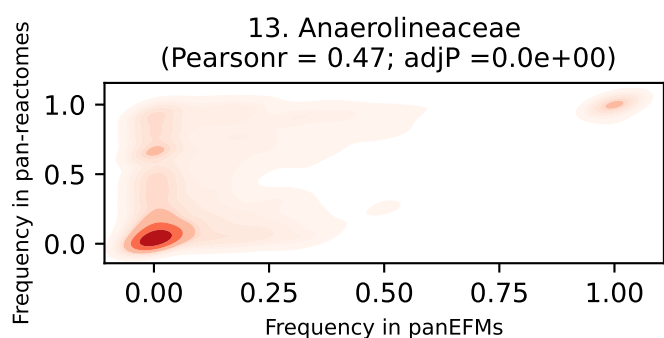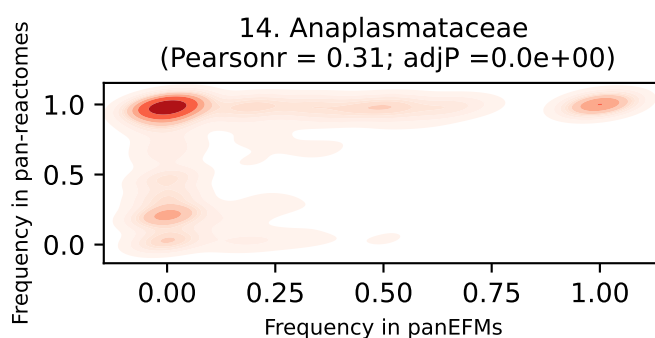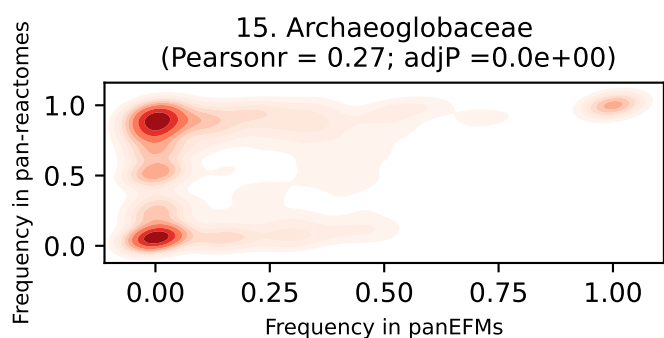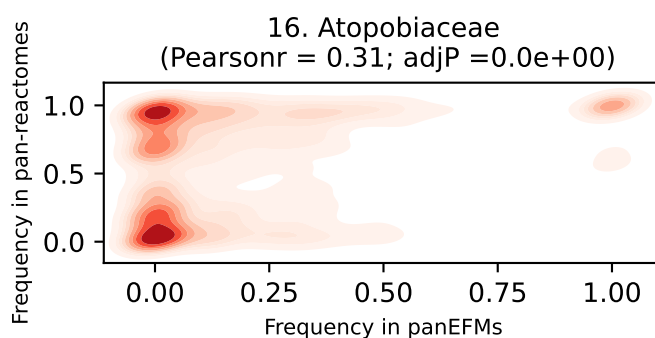

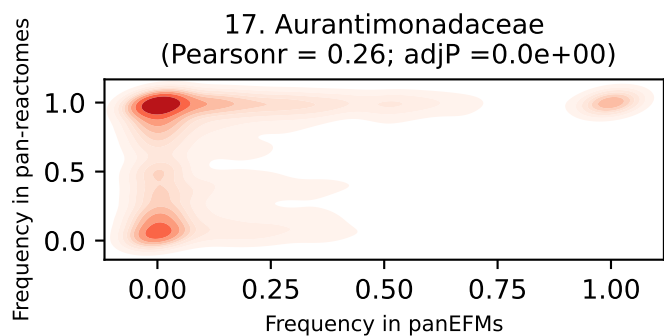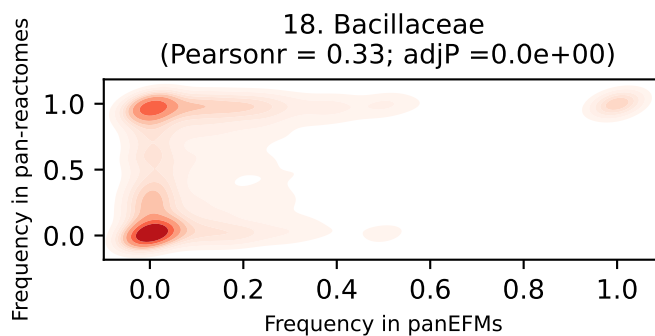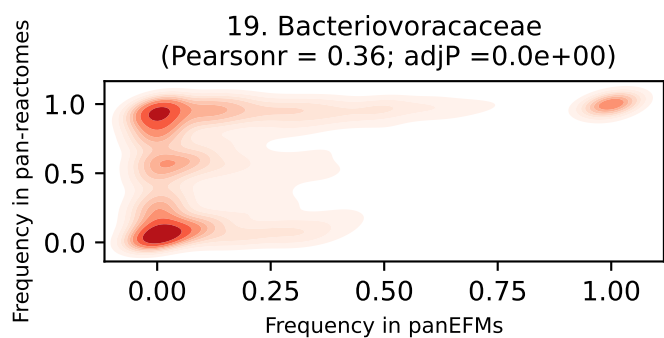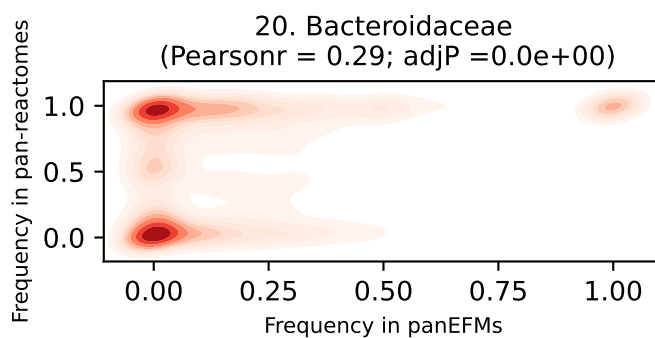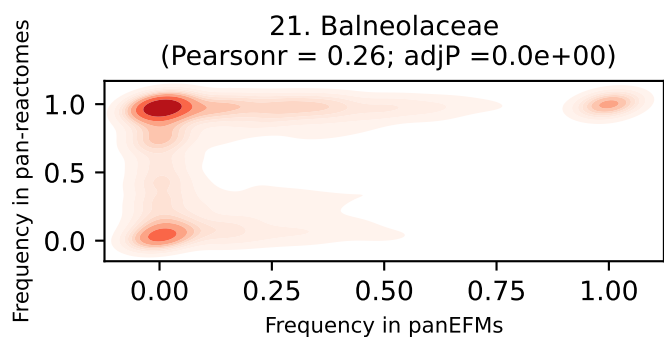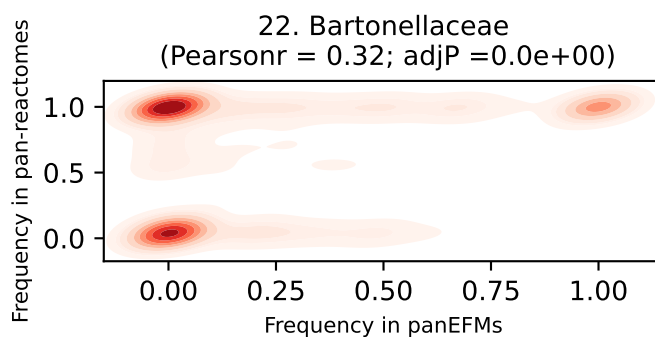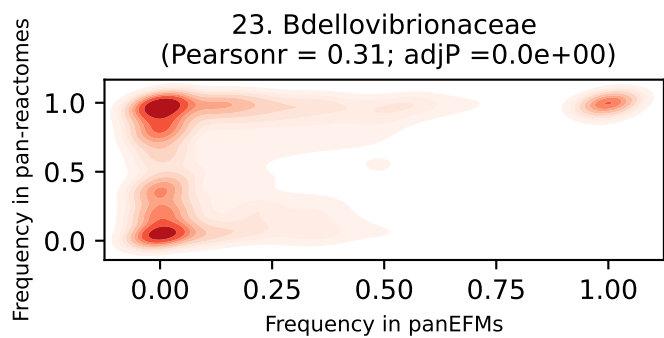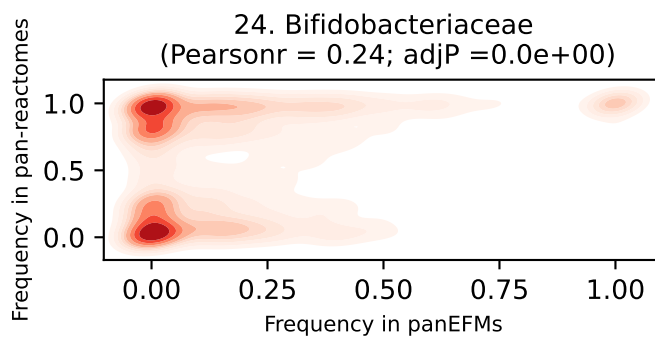

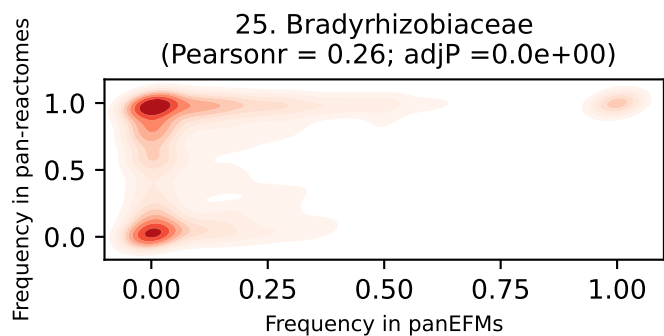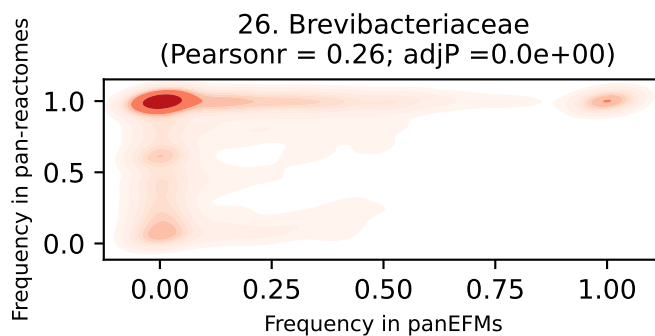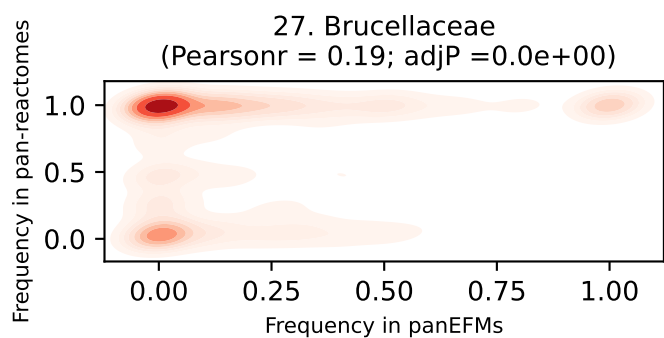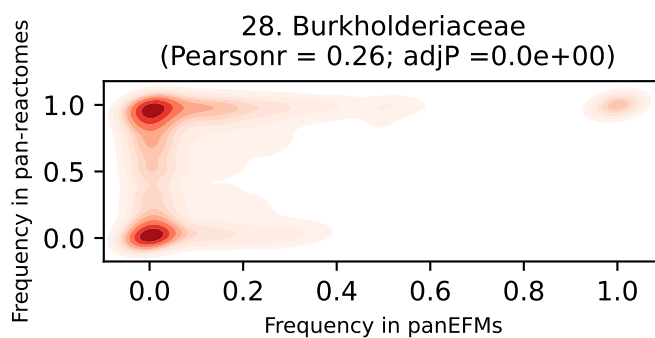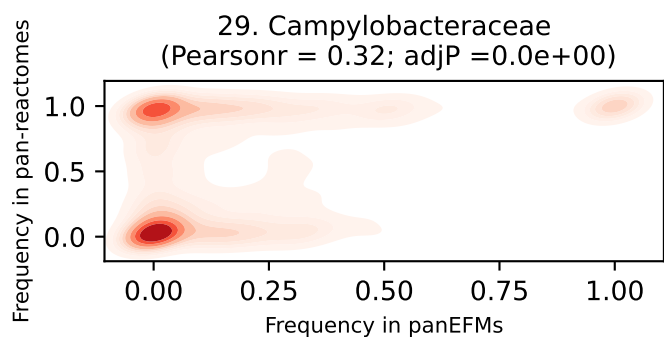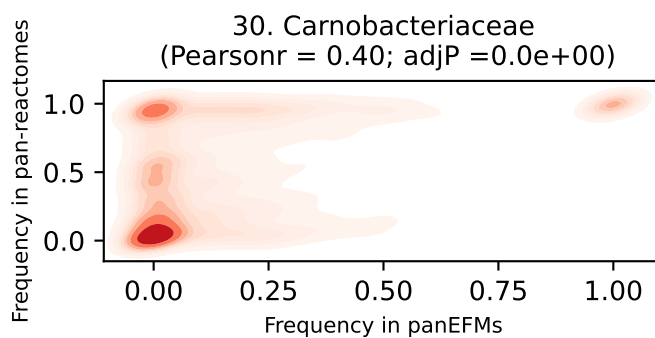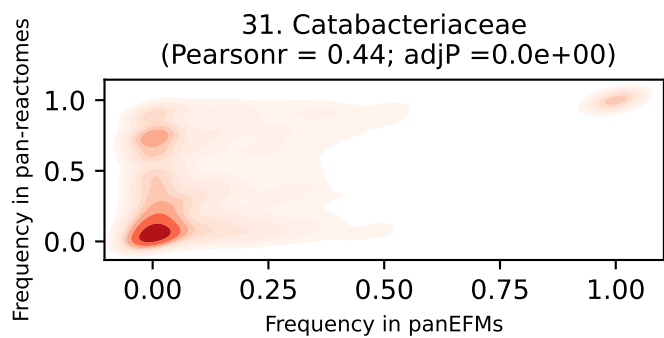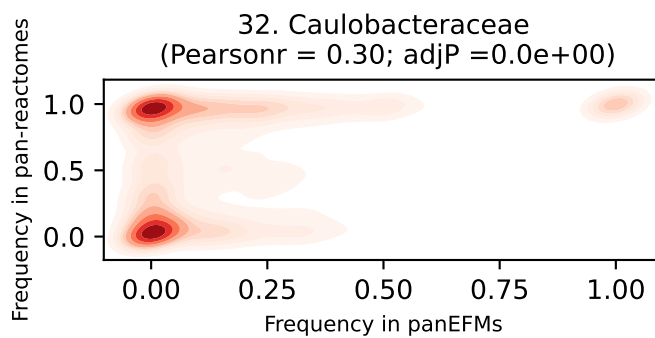

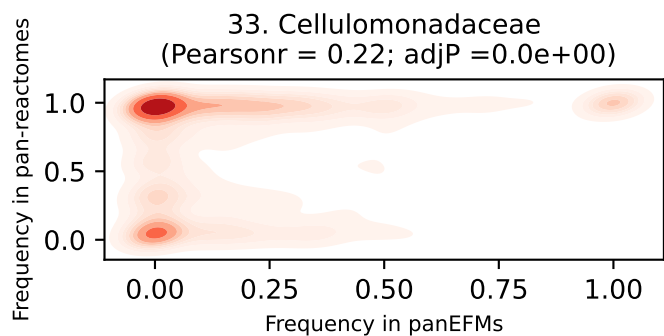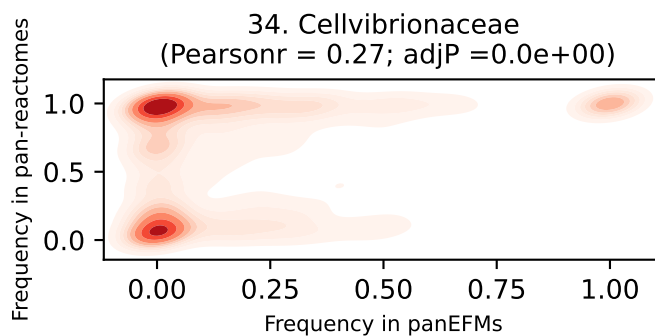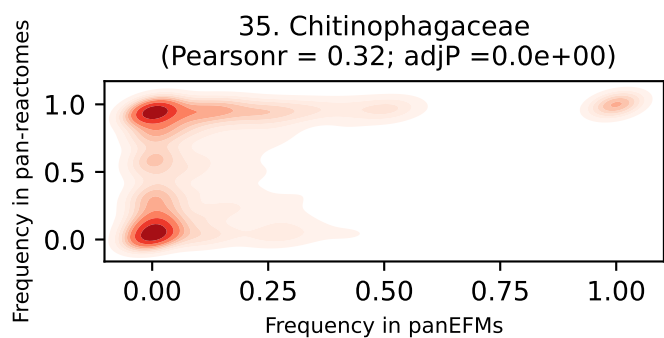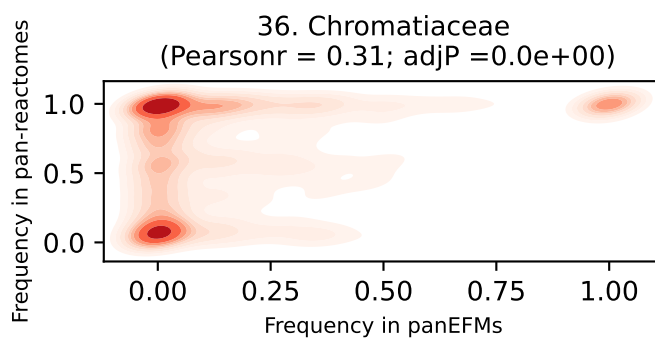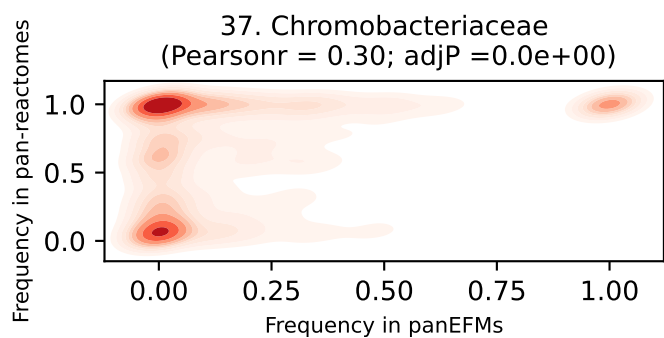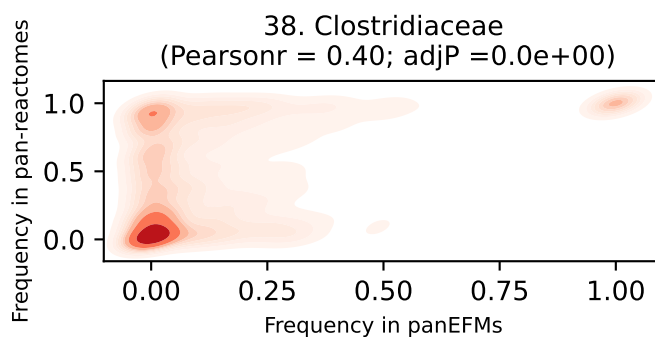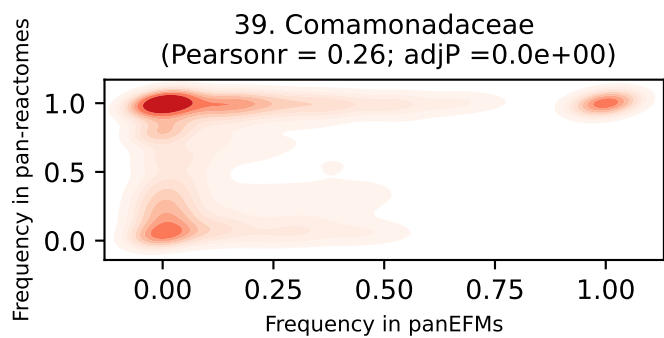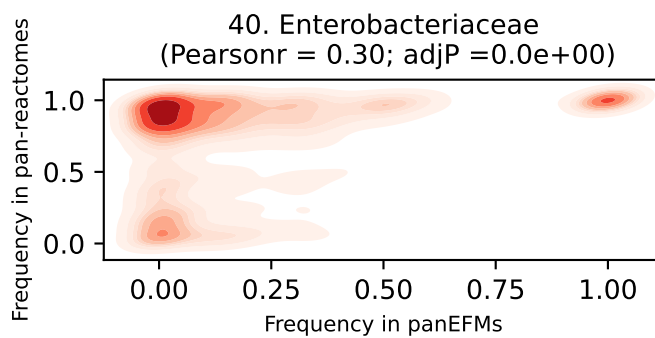

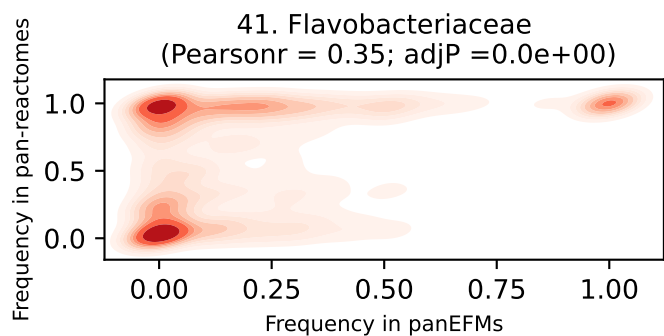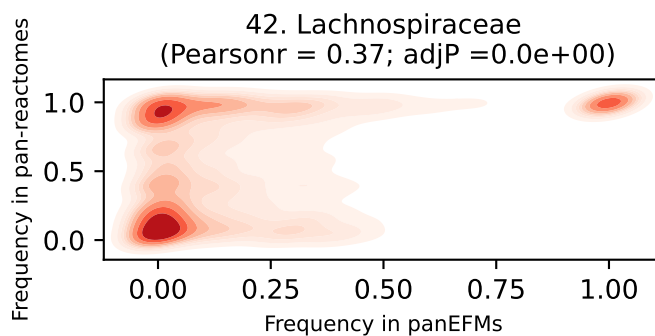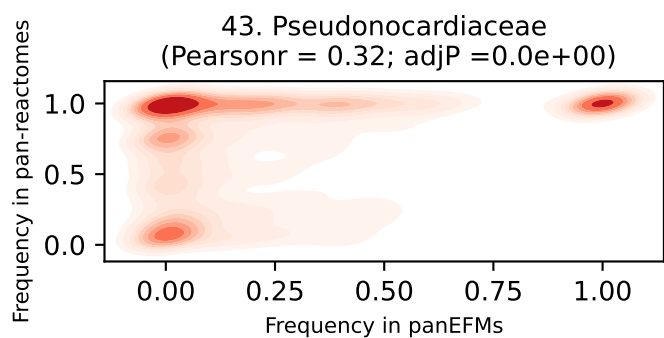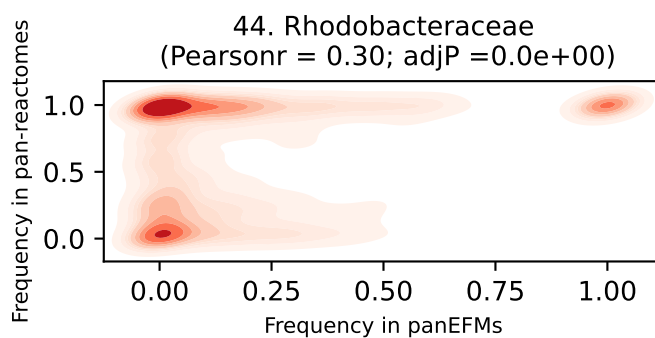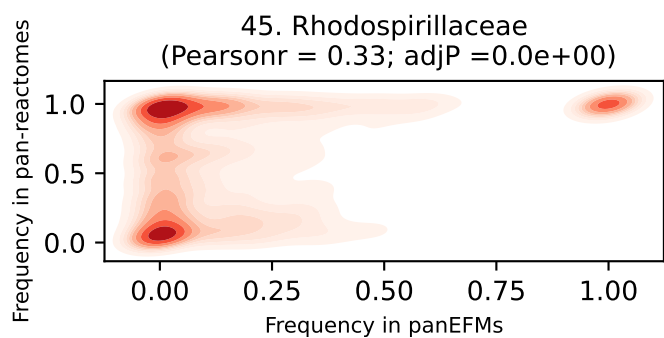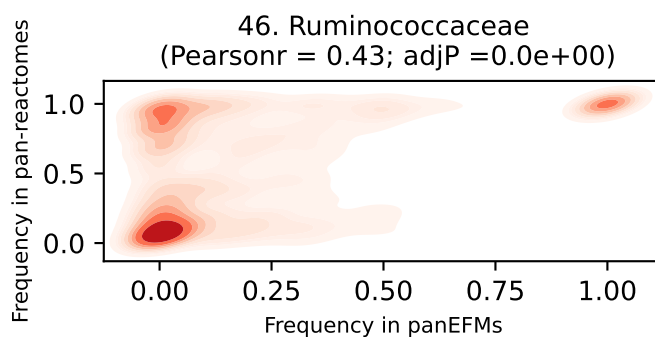

Supplement: Supplementary file 2 — Additional file 2: Figure S2. Distribution of pan-reactome reaction frequencies and panEFMs reaction frequencies. The x-axis contains the frequency of reactions in sampled panEFMs across random environments, while the y-axis contains the natural frequency coded by the genomes of the taxonomic families. [file 12862_2022_2052_MOESM2_ESM.pdf]

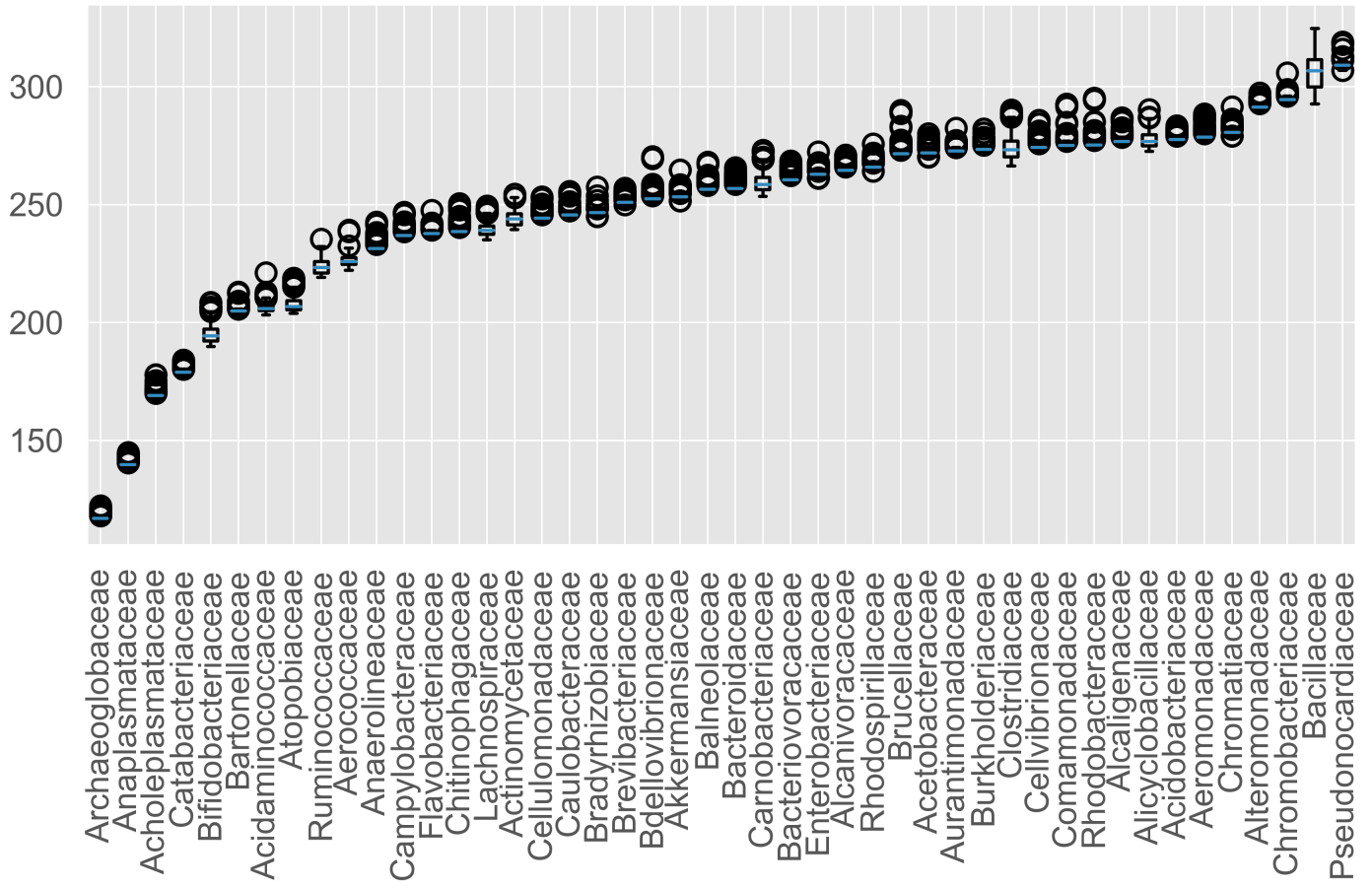

Supplement: Supplementary file 5 — Additional file 5: Figure S5. Size distribution of panEFMs sampled across random virtual environments. [file 12862_2022_2052_MOESM5_ESM.pdf]
