# Supplementary material for: Nutrition or nature: using elementary flux modes to disentangle the complex forces shaping prokaryote pan-genomes
Source: BMC Ecol Evol. 2022 Aug 16;22:101. doi: 10.1186/s12862-022-02052-3 (PMC9382767; doi:10.1186/s12862-022-02052-3)
Supplement: Supplementary file 3 — Additional file 3: Figure S3. Distribution of the environment-driven reaction score (EDS) and panEFMs reaction frequencies. [file 12862_2022_2052_MOESM3_ESM.pdf]

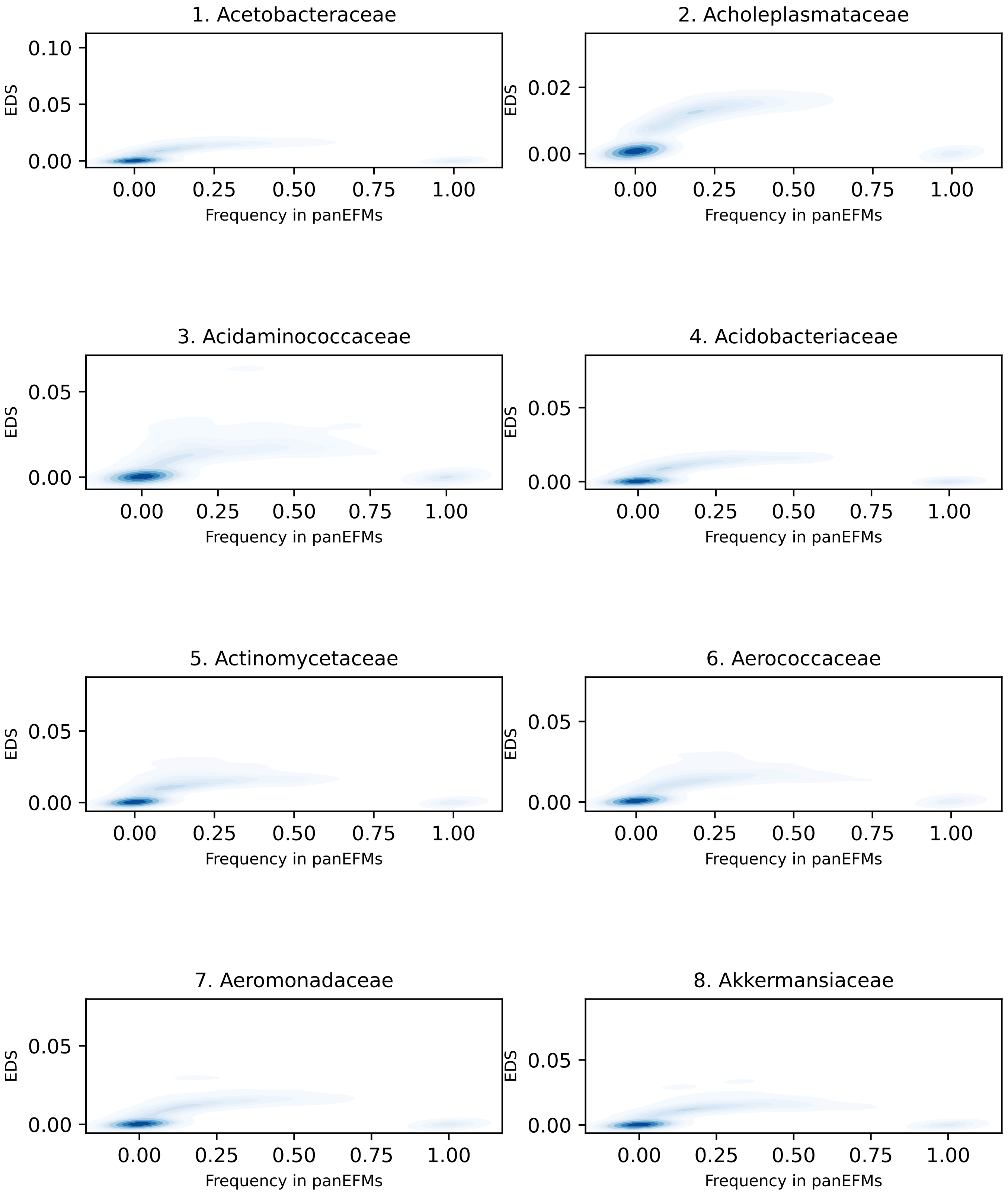
1. Acetobacteraceae
2. Acholeplasmataceae
3. Acidaminococcaceae
4. Acidobacteriaceae
5. Actinomycetaceae
6. Aerococcaceae
7. Aeromonadaceae
8. Akkermansiaceae
EDS
Frequency in panEFMs
0.00
0.05
0.10
0.02
0.25
0.50
0.75
1.00

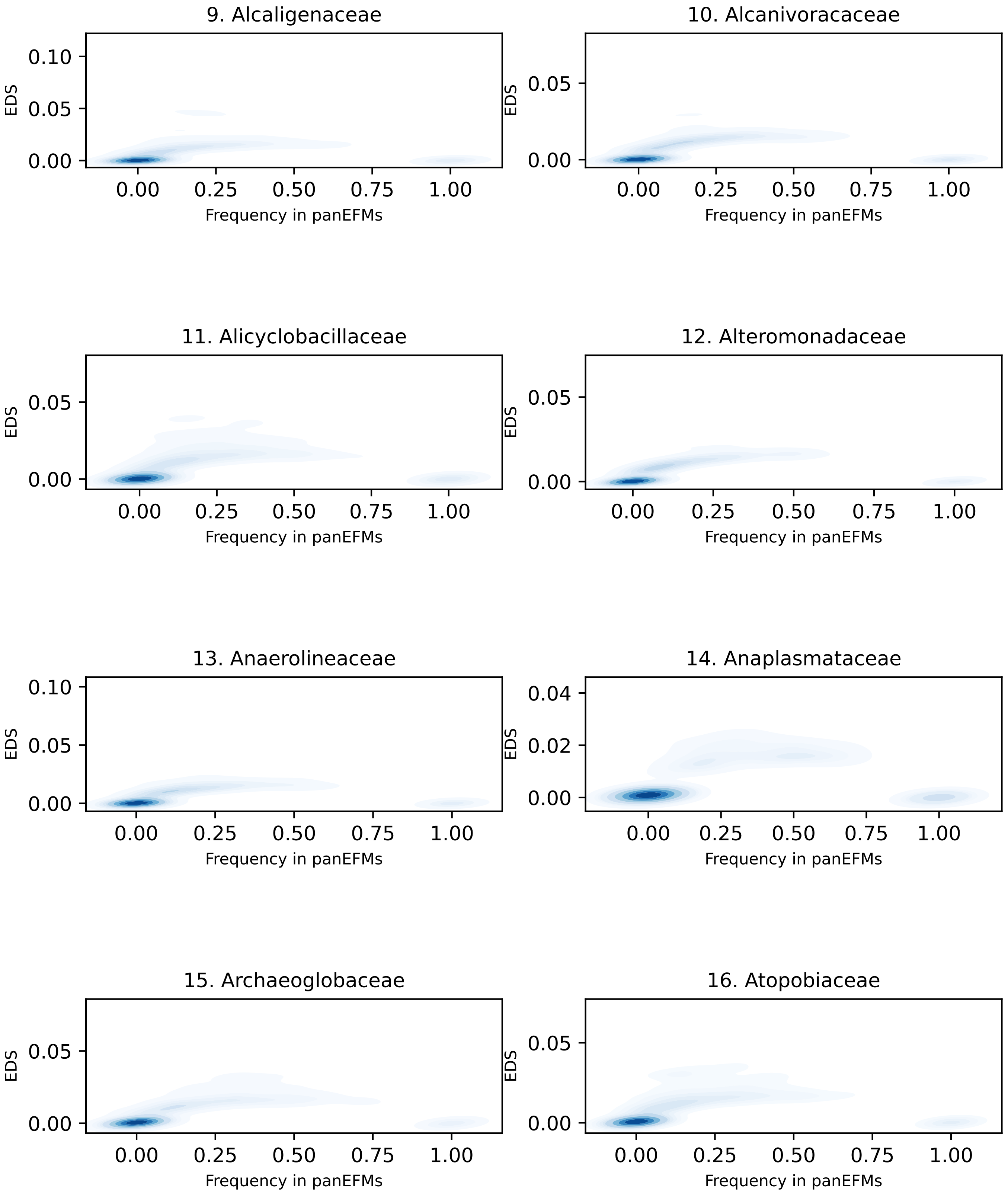
9. Alcaligenaceae
10. Alcanivoracaceae
11. Alicyclobacillaceae
12. Alteromonadaceae
13. Anaerolineaceae
14. Anaplasmataceae
15. Archaeoglobaceae
16. Atopobiaceae
EDS
Frequency in panEFMs
0.00
0.25
0.50
0.75
1.00
0.05
0.10
0.02
0.04

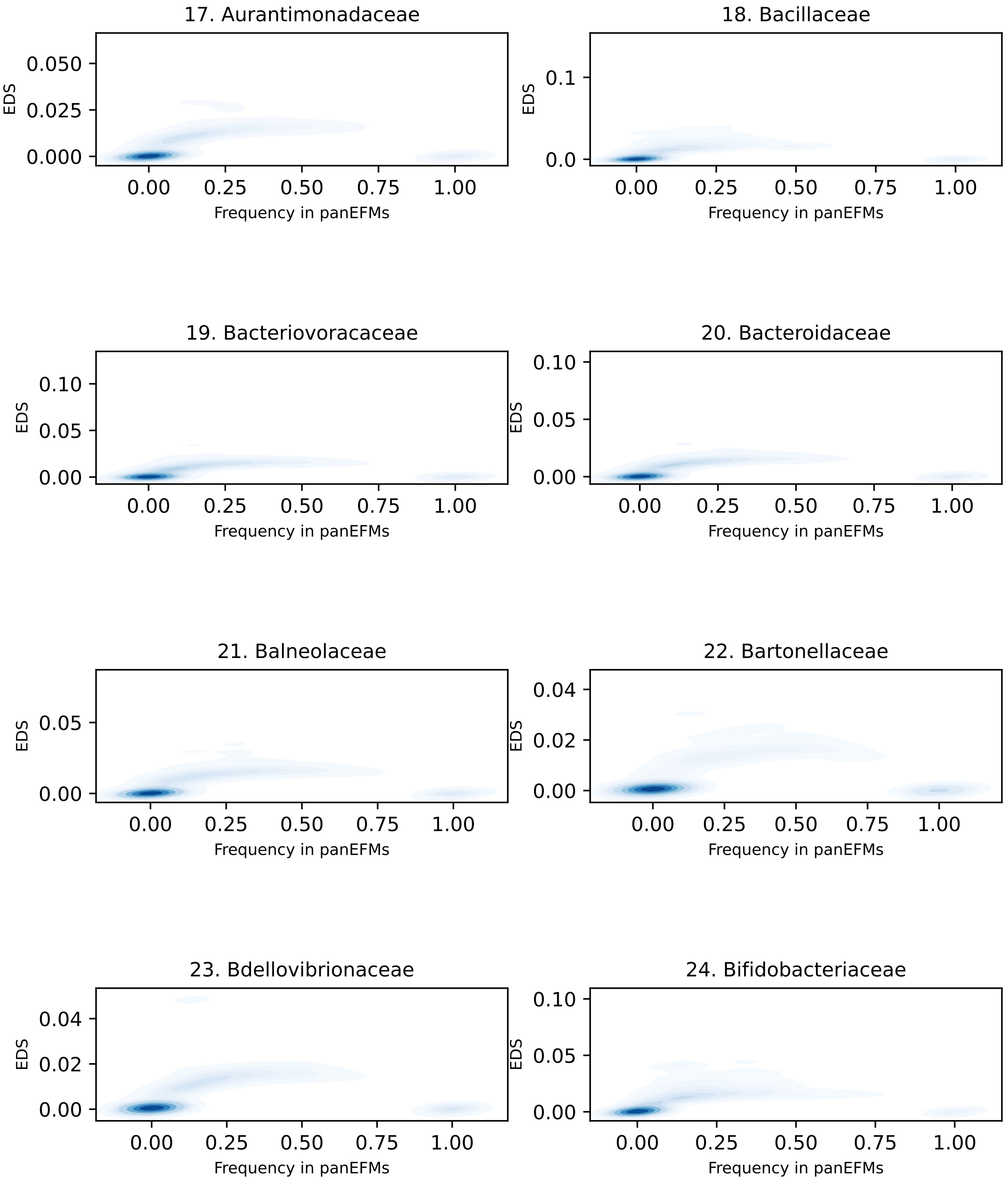

17. Aurantimonadaceae
0.050
0.025
0.000
EDS
0.00
0.25
0.50
0.75
1.00
Frequency in panEFMs
18. Bacillaceae
0.1
0.0
EDS
0.00
0.25
0.50
0.75
1.00
Frequency in panEFMs
19. Bacteriovoracaceae
0.10
0.05
0.00
EDS
0.00
0.25
0.50
0.75
1.00
Frequency in panEFMs
20. Bacteroidaceae
0.10
0.05
0.00
EDS
0.00
0.25
0.50
0.75
1.00
Frequency in panEFMs
21. Balneolaceae
0.05
0.00
EDS
0.00
0.25
0.50
0.75
1.00
Frequency in panEFMs
22. Bartonellaceae
0.04
0.02
0.00
EDS
0.00
0.25
0.50
0.75
1.00
Frequency in panEFMs
23. Bdellovibrionaceae
0.04
0.02
0.00
EDS
0.00
0.25
0.50
0.75
1.00
Frequency in panEFMs
24. Bifidobacteriaceae
0.10
0.05
0.00
EDS
0.00
0.25
0.50
0.75
1.00
Frequency in panEFMs

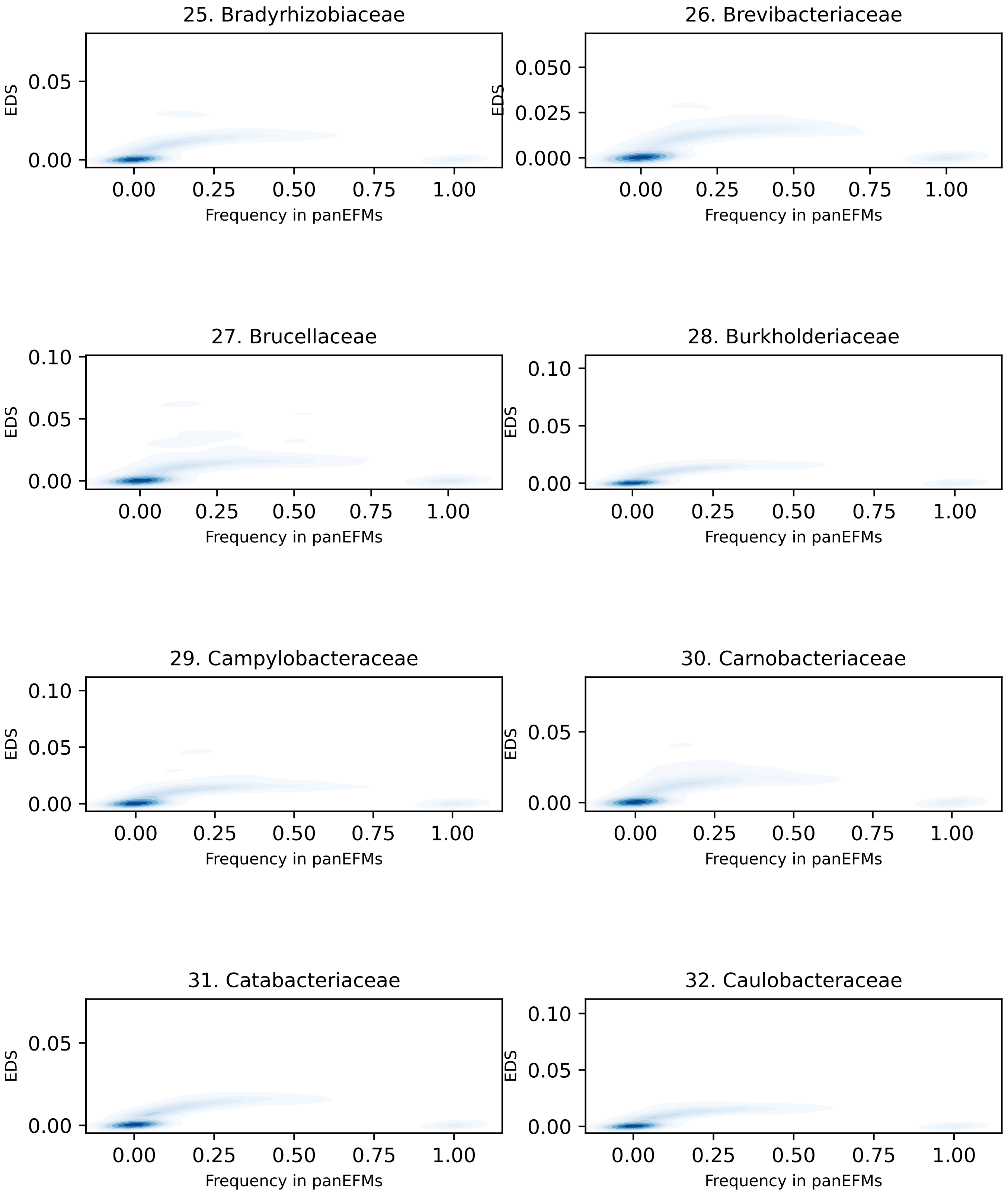
25. Bradyrhizobiaceae
26. Brevibacteriaceae
27. Brucellaceae
28. Burkholderiaceae
29. Campylobacteraceae
30. Carnobacteriaceae
31. Catabacteriaceae
32. Caulobacteraceae
EDS
Frequency in panEFMs
0.00
0.25
0.50
0.75
1.00
0.05
0.10
0.000
0.025
0.050

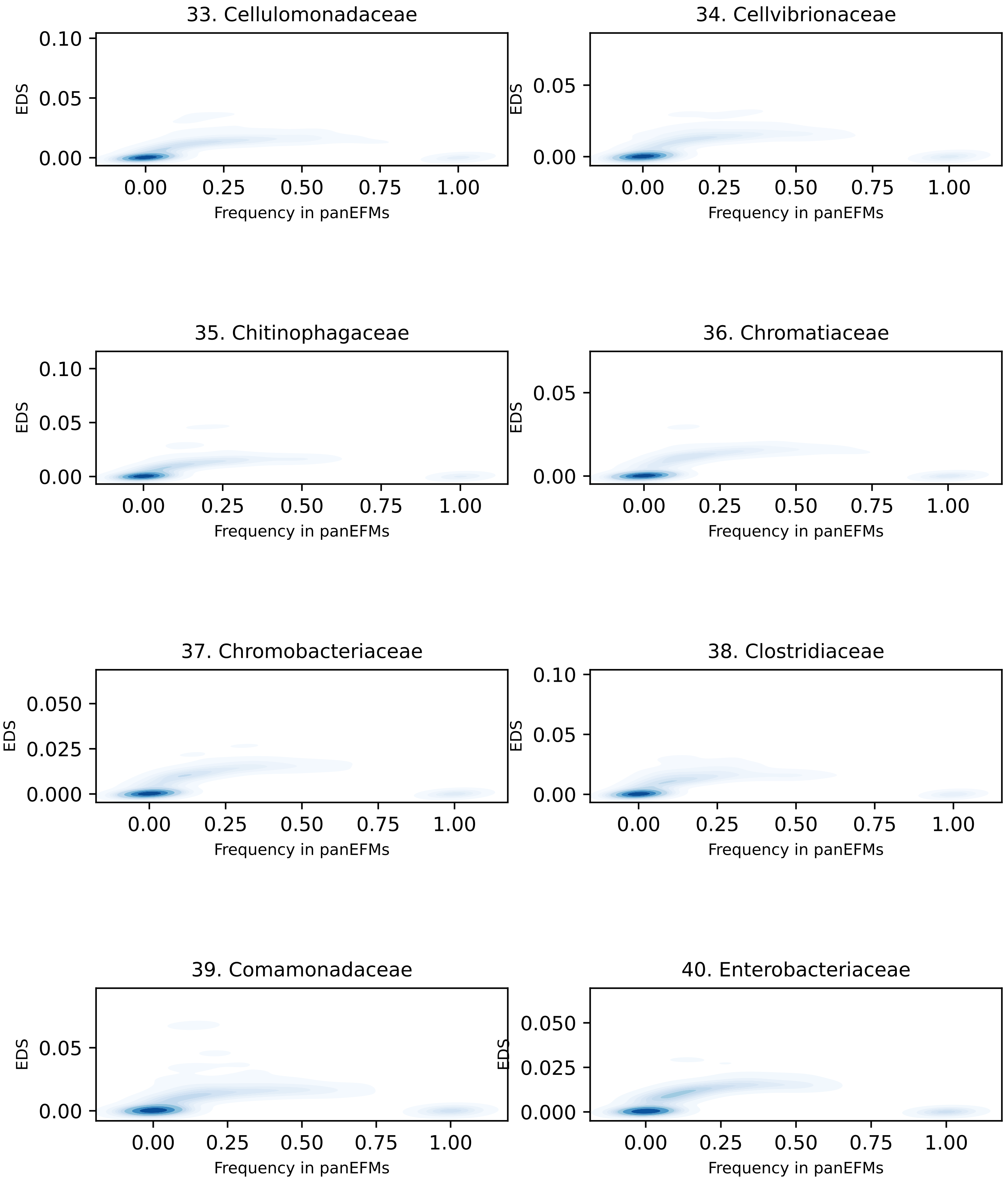
33. Cellulomonadaceae
34. Cellvibrionaceae
35. Chitinophagaceae
36. Chromatiaceae
37. Chromobacteriaceae
38. Clostridiaceae
39. Comamonadaceae
40. Enterobacteriaceae
EDS
Frequency in panEFMs
0.00
0.25
0.50
0.75
1.00
0.05
0.10
0.000
0.025
0.050

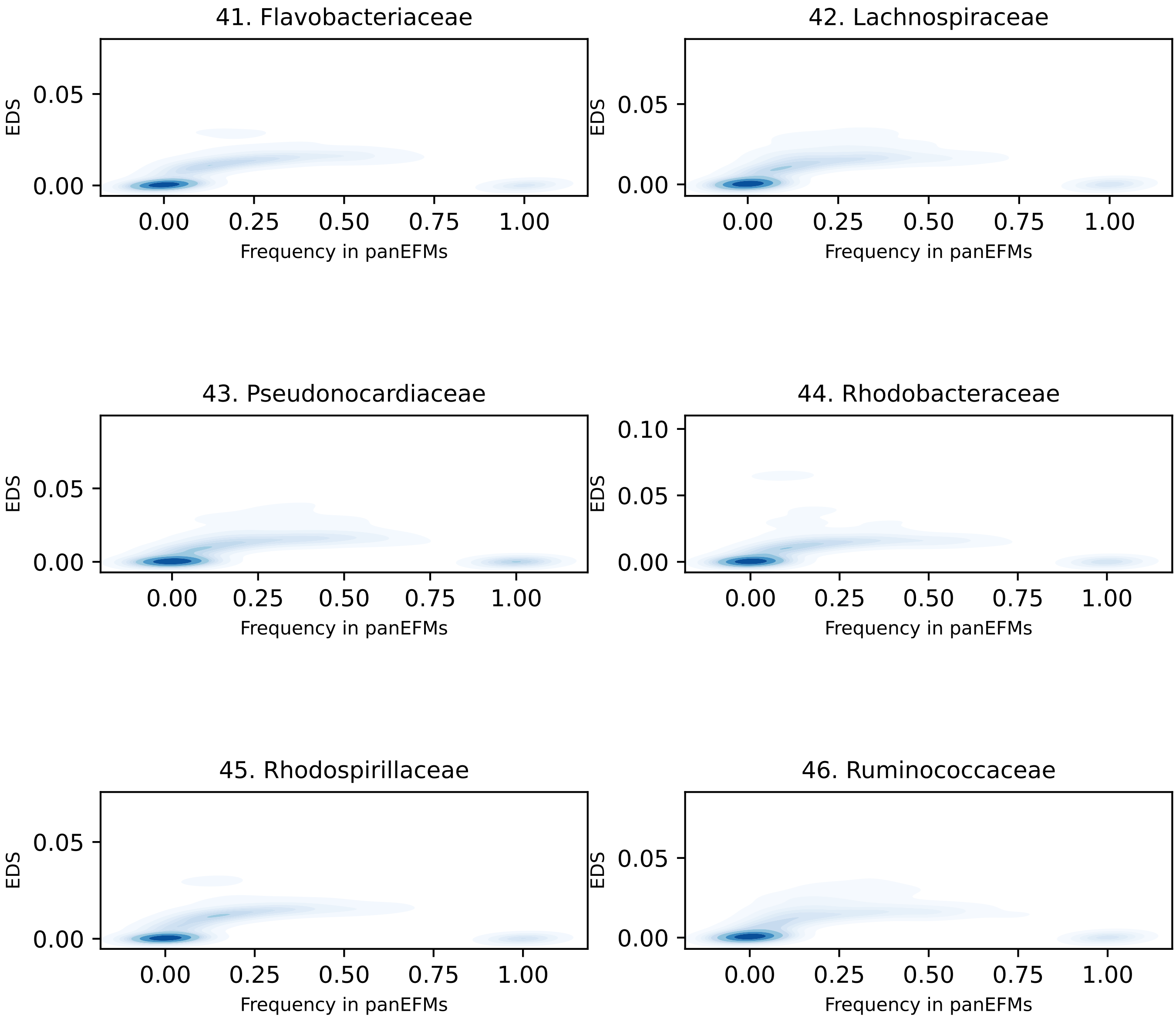
41. Flavobacteriaceae
42. Lachnospiraceae
43. Pseudonocardiaceae
44. Rhodobacteraceae
45. Rhodospirillaceae
46. Ruminococcaceae
EDS
Frequency in panEFMs
0.00
0.05
0.10
0.25
0.50
0.75
1.00
