# Supplementary material for: Nutrition or nature: using elementary flux modes to disentangle the complex forces shaping prokaryote pan-genomes
Source: BMC Ecol Evol. 2022 Aug 16;22:101. doi: 10.1186/s12862-022-02052-3 (PMC9382767; doi:10.1186/s12862-022-02052-3)
Supplement: Supplementary file 4 — Additional file 4: Figure S4. Comparison of panEFMs across environments. Scatter plot of the average reaction frequency of panEFMs defined across random virtual environments and within each environment. [file 12862_2022_2052_MOESM4_ESM.pdf]

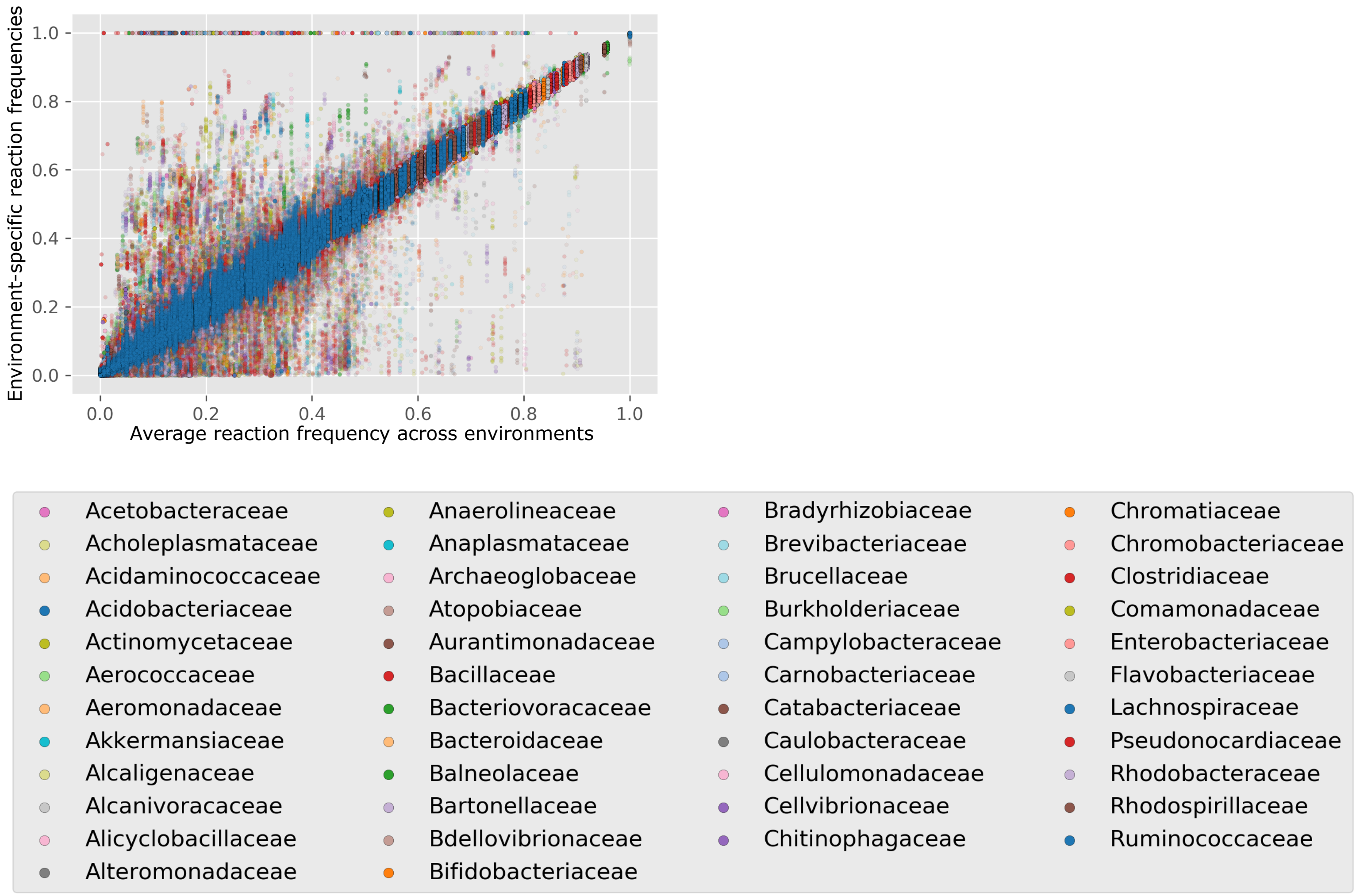
Environment-specific reaction frequencies
1.0
0.8
0.6
0.4
0.2
0.0
0.0
0.2
0.4
0.6
0.8
1.0
Average reaction frequency across environments
Acetobacteraceae
Acholeplasmataceae
Acidaminococcaceae
Acidobacteriaceae
Actinomycetaceae
Aerococcaceae
Aeromonadaceae
Akkermansiaceae
Alcaligenaceae
Alcanivoracaceae
Alicyclobacillaceae
Alteromonadaceae
Anaerolineaceae
Anaplasmataceae
Archaeoglobaceae
Atopobiaceae
Aurantimonadaceae
Bacillaceae
Bacteriovoracaceae
Bacteroidaceae
Balneolaceae
Bartonellaceae
Bdellovibrionaceae
Bifidobacteriaceae
Bradyrhizobiaceae
Brevibacteriaceae
Brucellaceae
Burkholderiaceae
Campylobacteraceae
Carnobacteriaceae
Catabacteriaceae
Caulobacteraceae
Cellulomonadaceae
Cellvibrionaceae
Chitinophagaceae
Chromatiaceae
Chromobacteriaceae
Clostridiaceae
Comamonadaceae
Enterobacteriaceae
Flavobacteriaceae
Lachnospiraceae
Pseudonocardiaceae
Rhodobacteraceae
Rhodospirillaceae
Ruminococcaceae
