# Supplementary material for: Nutrition or nature: using elementary flux modes to disentangle the complex forces shaping prokaryote pan-genomes
Source: BMC Ecol Evol. 2022 Aug 16;22:101. doi: 10.1186/s12862-022-02052-3 (PMC9382767; doi:10.1186/s12862-022-02052-3)
Supplement: Supplementary file 6 — Additional file 6: Table S1. Reactions in the toy model [file 12862_2022_2052_MOESM6_ESM.pdf]

Table S1: reactions in the toy model.

Reaction	Equation
R1	M1_e →M1_i + M2_i
R2	M2_e → M1_i + M3_i
R3	M3_e → M4_i
R4	M4_e → M5_i
R5	M5_e → M6_i
R6	M6_e + M7_e → M7_i
R7	M8_e → M8_i
R8	M9_e → M8_i
R9	M1_i → M9_1
R10	M2_i → M10_e
R11	M7_i → M8_i
R12	M8_i + M6_i → M10_i
R13	M5_i → M11_i
R14	M3_i + M9_i → M12_i
R15	M4_i → M5_i + M3_i
Biomass	M10_i + M11_i + M12_12
